# Re-Visiting Phylogenetic and Taxonomic Relationships in the Genus *Saga* (Insecta: Orthoptera)

**DOI:** 10.1371/journal.pone.0042229

**Published:** 2012-08-10

**Authors:** Balázs Kolics, Zoltán Ács, Dragan Petrov Chobanov, Kirill Márk Orci, Lo Shun Qiang, Balázs Kovács, Előd Kondorosy, Kincső Decsi, János Taller, András Specziár, László Orbán, Tamás Müller

**Affiliations:** 1 Department of Plant Sciences and Biotechnology, Georgikon Faculty, University of Pannonia, Keszthely, Hungary; 2 Fitolab Ltd., Budapest, Hungary; 3 Institute of Biodiversity and Ecosystem Research, Bulgarian Academy of Sciences, Sofia, Bulgaria; 4 Ecology Research Group of the Hungarian Academy of Sciences, Eötvös Loránd University and Hungarian Natural History Museum, Budapest, Hungary; 5 Reproductive Genomics, Strategic Research Program, Temasek Life Sciences Laboratory, National University of Singapore, Singapore, Singapore; 6 Department of Biological Sciences, National University of Singapore, Singapore, Singapore; 7 Department of Aquaculture, Faculty of Agricultural and Environmental Sciences, Szent István University, Gödöllő, Hungary; 8 Regional University Center of Excellence in Environmental Industry Based on Natural, Szent István University, Gödöllő, Hungary; 9 Department of Animal Sciences and Animal Breeding, Georgikon Faculty, University of Pannonia, Keszthely, Hungary; 10 Balaton Limnological Institute, Centre for Ecological Research, Hungarian Academy of Sciences, Tihany, Hungary; University of Guelph, Canada

## Abstract

Twelve of the 13 bushcricket species of the *Saga* genus are bisexuals and diploids, except the parthenogenetic and tetraploid bush cricket, *Saga pedo*. Despite a continuous research effort stretching through the 1900s, the taxonomic relationships of the *Saga* species are still disputed. In this study, our primary aim was to reveal natural relationships of the European *Saga* species and three of their Asian relatives, with special attention to the problematic taxonomy of two subspecies: *S. campbelli campbelli* and *S. c. gracilis*. Following a phylogenetic analysis of eight species, a comprehensive study was carried out on the above three taxa by using acoustic and morphometric approaches in parallel. Our phylogenetic data showed that European *Saga* species evolved from a monophyletic lineage. The geographical transitional specie*s S. cappadocica* was positioned between European and Asian lineages supporting the idea that the European *Saga* lineage originated phylogeographically from the Asian clade. The above results showed better agreement with the morphological data than with earlier ones based either on karyology or acoustic information only. After reviewing our data, we concluded that *Saga pedo* has most likely evolved from *S. c. gracilis* and not from *S. rammei* or *S. ephippigera*, as proposed by earlier studies. *S. c. gracilis* shares the same ITS2 haplotype with *S. pedo*, indicating that the latter could have evolved from populations of the former, probably through whole genome duplication. Based on acoustic and morphometric differences, we propose to elevate the two subspecies, *S. campbelli campbelli* and *S. c. gracilis,* to species level status, as *Saga gracilis* Kis 1962, and *Saga campbelli* Uvarov 1921. The present work sets the stage for future genetic and experimental investigations of *Saginae* and highlights the need for additional comprehensive analysis involving more Asian *Saga* species.

## Introduction

**Figure 1:**
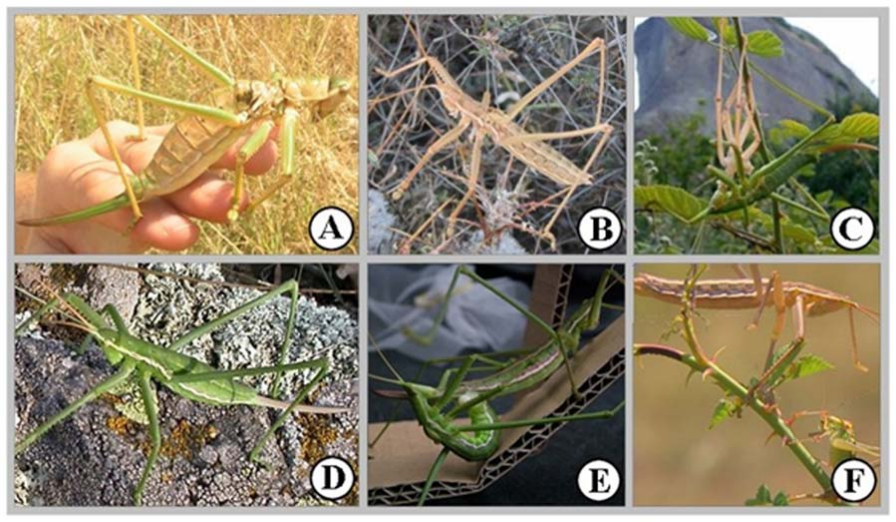
The European *Saga* species. A) *S. natoliae*; B) *S. rammei*; C) *S. hellenica*; D) *S. pedo*; E) *S. c. campbelli*; F) *S. c. gracilis.*

Orthoptera species comprise mostly herbivores and omnivores with few carnivorous representatives. Among the latter, subfamily *Saginae* includes some of the most specialised and the largest obligatory carnivorous bush crickets. The subfamily includes four genera, distributed over two highly separated regions - the South and Southeast of the Sub-Saharan Africa (three genera) and part of the Western Palearctic (genus *Saga*). Comprising the largest European orthopterans, the genus *Saga* contains 13 species [1], of which five inhabit Continental Europe (one of them penetrating into Western Siberia), while the rest live in Asia (the Caucasus region, Turkey, Syria, Lebanon, Israel, Iran, and Iraq). The majority of the *Saga* species are bisexual anddiploid; they inhabit the Balkan Peninsula and parts of the Middle East (see Fig. 1 for pictures of the European species concerned, and Fig. S1 for distribution of the species studied here). The only tetraploid member of the genus is the parthenogenetic bush cricket, *Saga pedo* Pallas 1771.It occupies a territory much larger than that of any bisexual species, from the coast of Portugal [2] to Xinjiang, Uyghur Region, China [3]. Moreover, it was also introduced to North-America [4].

The parthenogenetic bush cricket is protected throughout its distribution area and it was evaluated as vulnerable for its sparse populations, and being flightless, decreased capability to escape from danger[IUCN Red List of Threatened Species; http://www.iucnredlist.org/apps/redlist/search]. Although the rest of the *Saga* species are not protected, they also live in isolated populations at low density, and mainly on warm, southern hills of middle mountains, as xerothermic insects [2].

The *Saga* species are stalk hunters moving mostly in dark, whilst in the daytime they tend to fade into the plantscape [2], [5]. Thus, it is rather hard to find these bush crickets in the field, especially the imagoes. Due to the low number of the specimen observed, and the similarity of species, the earliest attempts to compile an identification key were not comprehensive [6], [7], [8]. There was “hardly any genus so hopelessly confused as the genus *Saga*” [9] until the middle of the last century when the first detailed description of the genus was established by Ramme [10]. Subsequently, an appropriate identification key was published by Kaltenbach [1].

Based on their morphology, Kaltenbach [1] outlined four *Saga* species groups as follows: A) *S. natoliae, S. rhodiensis, S. ephippigera*; B) *S. cappadocica, S. campbelli, S. rammei, S. hellenica;* C) *S. ornata*; D) *S. pedo* (only the species analyzed in our study are mentioned here). According to him, the place of origin of *S. pedo* was the area between the Black Sea and Caspian Sea.

Most of the orthopteran species produce sound; the main function of these signals is to help pair formation between conspecific males and females (see e.g. [11], [12], [13], [14]). Closely related and morphologically less differentiated species often produce conspicuously differentiated acoustic signals, which therefore can be used efficiently for examining species-level taxonomic problems [15], [16], [17], [18]. The songs of three European *Saga* species (*S. natoliae, S. hellenica* and *S. campbelli*) were first analyzed by Heller [19], whereas those of *S. rammei* together with a basic level comparison on five European taxa (*S. campbelli campbelli, S. campbelli gracilis, S. natoliae,* and *S. hellenica*) were provided by Kolics and colleagues [20]. The grouping based on sonometric parameters of male calling songs is partly supported by the one based on chromosome information raised by Warchałowska-Śliwa and coworkers [21]. Interestingly, differences were found in the calling song of the two subspecies of *S. campbelli*: *S. c. campbelli* and *S. c. gracilis* [20].

The chromosome sets of the *Saga* species and subspecies analysed here were assessed earlier by several authors [21], [22], [23], [24], [25], [26], [27], [28]. Lemonnier-Darcemont and colleagues [23] made the first attempt to define the phylogenic positions of *S. pedo*, *S.campbelli*, *S. rammei,S. natoliae, S. hellenica*, *S. ornata,* and *S. cappadocica* based on chromosomal rearrangements. According to them, *S. pedo* originated from *S. rammei* by tetraploidisation, and the most basal species were *S. ephippigera* and *S. cappadocica*. Warchałowska-Śliwa and colleagues [21] proposed the following species groups based on the chromosome number: A) *S. ephippigera, S. ornata, S. cappadocica*; B) *S. natoliae*, *S. rhodiensis*, *S. hellenica; *C) *S. campbelli*; D) *S. rammei*; and E) *Saga pedo*. The most basal species were thought to be those in group “A”. The parthenogenetic bush cricket was shown to bear a karyotype of 4n = 68, possibly derived from that of *S. ephippigera* [21]. On the other hand, according to Dutrillaux and colleagues [29] the karyotype of *S. pedo* is 5n = 70 (but see Discussion), and the “karyotype of *S. pedo*, with 10–11 metacentric chromosomes could be derived from that of species like *S. campbelli* or *S. rammei*, however, 4–5 submetacentrics would remain a mystery”.

Despite all the knowledge obtained from the above studies, natural relationships within the genus *Saga* are still uncertain. Many questions regarding the taxonomic status of several species and their phylogenetic connections are waiting to be answered. The phylogeny and evolution of the parthenogenetic *S. pedo* are especially interesting, since this species reproduces asexually and occupies a territory larger than the combined range of all the other species in this genus. Furthermore, there is a report on the existence of a male parthenogenetic bush cricket [30]. Hybrids (*S. rammei* ♂×*S. pedo ♀*)obtained from matings in captivity were also described, based on their morphometric characterisation [31].

The aim of the present work was to determine the evolutionary relationships between the European *Saga* species, and to shed light on the likely origin of the most peculiar species of the genus, the parthenogenetic bush cricket. We have analyzed the phylogenetic relationships of European *Saga* representatives (with limited comparisons to Asian ones) by using mitochondrial and nuclear DNA sequences. We have also performeda comparative morphological study on three taxa (*S. c. campbelli*, *S. c. gracilis* and *S. pedo*). Additionally, we have also compared the calling songs of the subspecies *S. c. campbelli* and *S. c. gracilis* (as *S. pedo* is the only mute species of the genus) by multivariate statistical methods.

## Materials and Methods

### 2.1. Sampling

The 27 *Saga* specimens representing nine species were collected in Central Europe (Hungary), the Balkan Peninsula (Macedonia, Bulgaria), Asia Minor (Turkey) and the Middle East (Israel) during the summers of 2006 and 2008 (for details see Table 1). Sample Seph09 originated from the area where the ranges of the two subspecies of *S. ephippigera, S. e. ephippigera* and *S. e. syriaca*, overlap, and the sample comprised only part of the abdominal tissue. Thus, it may represent either of the two subspecies.

**Table 1:** Details of sample collection.

Taxon	Code	Locality	Accession Numbers			
			*cytb*	*cox*I	16S rRNA	ITS2
*S. cappadocica*	Scap01	Turkey, Cappadocia, Avanos	GU206270	GU206243	GU206296	GU206323
*S. cappadocica*	Scap02	Turkey, Cappadocia, Avanos	GU206271	GU206244	GU206297	GU206324
*S. c. campbelli*	Scac03	Bulgaria, Gorna Breznitsa, Maleshevska Mt.	GU206272	GU206245	GU206298	GU206325
*S. c. campbelli*	Scac04	Bulgaria, Gorna Breznitsa, Maleshevska Mt.	GU206273	GU206246	GU206299	GU206326
*S. c. campbelli*	Scac05	Bulgaria, Gorna Breznitsa, Maleshevska Mt.	GU206274	GU206247	GU206300	GU206327
*S. c. gracilis*	Scag06	Bulgaria, Plevoun, East Rhodope Mts., Surta ridge	GU206275	GU206248	GU206301	GU206328
*S. c. gracilis*	Scag07	Bulgaria, Plevoun, East Rhodope Mts., Surta ridge	GU206276	GU206249	GU206302	GU206329
*S. c. gracilis*	Scag08	Bulgaria, Plevoun East Rhodope Mts., Surta ridge	GU206277	GU206250	GU206303	GU206330
*S. ephippigera*	Seph09	Turkey, Adiaman-Malatya provinces border, Nemrut summit	-	GU206251	GU206304	-
*S. ephippigera*	Seph10	Israel, Golan Heights	GU206278	GU206252	GU206305	GU206331–32
*S. hellenica*	Shel11	Macedonia, Gorno Nerezi, Vodno Mt.	GU206279	GU206253	GU206306	GU206333
*S. natoliae*	Snat12	Bulgaria, Roupite, Kozhouh hill, Strouma valley	GU206280	GU206254	GU206307	GU206334
*S. natoliae*	Snat13	Macedonia, Bogoslovec, Ovce Pole region	GU206281	GU206255	GU206308	GU206335
*S. natoliae*	Snat14	Macedonia, Bogoslovec, Ovce Pole region	GU206282	GU206256	GU206309	GU206336
*S. pedo*	Sped15	Hungary, Nagykovácsi, Budai Mt.	GU206283	GU206257	GU206310	GU206337
*S. pedo*	Sped16	Hungary, Nagykovácsi, Budai Mt.	GU206284	GU206258	GU206311	GU206338
*S. pedo*	Sped17	Hungary, Zalaszántó, Keszthely Mt.	GU206285	GU206259	GU206312	GU206339
*S. pedo*	Sped18	Hungary, Zalaszántó, Keszthely Mt.	GU206286	GU206260	GU206313	GU206340
*S. pedo*	Sped19	Hungary, Zalaszántó, Keszthely Mt. (Own rearing)	GU206287	GU206261	GU206314	GU206341
*S. pedo*	Sped20	Bulgaria, Kavarna, N Black Sea coast, Bolata valley	GU206288	GU206262	GU206315	GU206342
*S. pedo*	Sped21	Bulgaria, Kavarna, N Black Sea coast, Bolata valley	GU206289	GU206263	GU206316	GU206343
*S. pedo*	Sped22	Bulgaria, Kavarna, N Black Sea coast, Bolata valley	GU206290	GU206264	GU206317	GU206344
*S. pedo*	Sped23	Macedonia, Lipova Livada pass, Galichica Mt.	GU206291	GU206265	GU206318	GU206345
*S. rammei*	Sram24	Macedonia, Berkirlija, Slan Dol plane	GU206292	GU206266	GU206319	GU206346
*S. rammei*	Sram25	Macedonia, Bogoslovec, Ovce Pole region	GU206293	GU206267	GU206320	GU206347
*S. rammei*	Sram26	Macedonia, Bogoslovec, Ovce Pole region	GU206294	GU206268	GU206321	GU206348
*S. ornata*	Sorn27	Israel, Golan Heights	GU206295	GU206269	GU206322	GU206349–50

### 2.2. DNA extraction, PCR amplification and sequencing of selected genes

Total DNA was extracted from the muscle of hind femur or tibia using the DNEasy Tissue Kit (QIAGEN, Hilden, Germany) following the manufacturer's protocol for insect DNA extraction. Fragments of three mitochondrial genes (433 bp from cytochrome *b* or *cytb*, 660 bp from cytochrome oxidase I, *cox*I, and 507–508 bp from 16S rRNA) and one nuclear gene (853–1379 bp from ITS2) were amplified and sequenced. Primers used for PCR and sequencing are listed in Table S1.

Polymerase chain reactions were carried out in 25 µL, and they contained 1 U Taq polymerase, 2.5 µl 10xTaq buffer (Fermentas, USA), 1.5 µl MgCl_2_(25 mM), 0.5 µl dNTPs (10 mM), 0.35 µl primers (10 pmol/µl), 1.0 µl template DNA (70–250 ng/µl) and 18.85 µl dH_2_O. PCR products were purified using shrimp alkaline phosphatase and *E. coli* exonuclease I. PCR products of *cytb*, *cox*I and 16S rRNA were sequenced directly using ABI BigDye Terminator chemistry on ABI automated sequencer (Applied Biosystems), and in both directions to minimize PCR artifacts, ambiguities and base calling errors. ITS2 PCR products resisted direct sequencing, revealing the existence of multiple fragments. Cloning of ITS2 fragments was carried out using pGEM-T Easy Vector (Promega, USA) cloning kit. Several ITS2 clones were sequenced from each sample (at least 10-fold coverage) to assign base calling. Cropping and cleaning of raw sequences were performed by BioEdit software [32]. All new sequences were deposited into GenBank (see accession numbers in Table 1).

### 2.3. Phylogenetic analysis

Multiple alignments of mitochondrial sequences were made using ClustalX v2.0.10 [33] with default parameters. ITS2 sequences were aligned by eye using the BioEdit software [32].All *cytb* and *cox*I genes were the same length (433 bp and 660 bp, respectively) and were checked for an open reading frame in order to exclude nuclear pseudogenes [34]. 16S rRNA mitochondrial sequences were of 507 bp length, except for *S. natoliae* samples (Snat12, Snat13 and Snat14) that were of 508 bp. The length of most of the ITS2 sequences was between 853–860 bp.However, *S. cappadocica* (Scap01, Scap02), *S. ephippigera* (Seph10) and *S. ornata* (Sorn27) samples contained longer sequences including a 115 bp insertion (Fig. S2). This insert region was removed prior to the phylogenetic analyses. In addition, *S. ephippigera* and *S. ornata* samples showed intra-individual ITS2 variability: both species carried two different haplotypes that were both included in the phylogenetic analyses after the removal of extra insertion sequences from the longer clones. For the calculation of genetic distance, matrix analyses were conducted using the Maximum Composite Likelihood method in MEGA4 [35]. Codon positions included were 1st+2nd+3rd+Noncoding. All positions containing gaps and missing data were eliminated from the dataset.

Phylogenetic analyses on DNA sequences were performed using maximum-parsimony (MP) and Bayesian Analyses approaches, using MEGA4 and MRBAYES 3.1, respectively [36]. Both approaches were executed for the nuclear ITS2 and mitochondrial DNA sequences (separated and concatenated *cox*I, *cytb*, 16S rRNA). For sample Seph09 (*S. ephippigera*) only *cox*I and 16S rRNA sequences were available due to partially degraded DNA source. Sequences of the conehead bush cricket *Banza unica* (Conocephalidae: Copiphorinae) were utilized as outgroup (GenBank accession numbers: DQ649501, DQ649525) for mitochondrial sequences, whereas no outgroup was used for the ITS2 trees in absence of a suitable candidate.

The MP trees were obtained using the Close-Neighbor-Interchange algorithm with search level 3 [35], [37] in which the initial trees were obtained with the random addition of sequences (20 replicates). The percentage of replicate trees in which the associated taxa clustered together in the bootstrap test (1000 replicates) were calculated [37]. All positions containing gaps and missing data were eliminated from the datasets.

Phylogenetic relationships were also estimated using Bayesian inference for sequence datasets. Datasets were partitioned (where applicable) by gene and codon position. Appropriate molecular models were selected by MRMODELTEST v2.3 [38]. GTR+I+G model of sequence evolution was applied to each partition. Phylogenies were constructed using individual genes and datasets of two (protein coding mitochondrial genes: *cytb* and *cox*I) or three genes (*cytb*, *cox*I, and 16S rRNA) with the gamma shape parameter, the proportion of invariant sites, base frequencies and substitution rates unlinked across all partitions. A typical starting point was a run length of 2 million generations, sample frequency of 100 generations and a burn in time of half million generations.

The Neighbour Joining tree base on the ITS2 insertion sequences was generated by MEGA4. The evolutionary distances used to infer the phylogenetic tree were computed using the Maximum Composite Likelihood method and are in the units of the number of base substitutions per site.

### 2.4. Analysis of calling songs

Sound recordings were made in our laboratory in Hungary between July 18^th^ and 30^th^ in the years 2006 and 2008, according to the method described earlier [20]. Air temperature varied between 26–27.2°C during song recordings. Calling songs (spontaneous songs produced by isolated males [17]) were examined for the following grouping: 1) Duration of echemes (DE) (an echeme is the first order grouping of syllables) was measured from the end of the first recognizable syllable (the sound produced by one opening-closing movement cycle of the tegmina) to the end of the last syllable (that measurement points were chosen to increase the repeatability of our measurements and minimise measurement errors, since syllables often have uncertain beginning with a slowly developing crescedo exceeding background noise level at a rather uncertain point. In contrast the end of syllables is quite clear. This way our data have a smaller amount of measurement error, however by that measurement method we systematically underestimate the real DE by 10–15 ms (the duration of the first syllable), but the cause less than 1% difference from real DE); 2) Echeme repetition period (ERP) was measured from the beginning of an echeme to the beginning of the next echeme. An additional derived song parameter used in this study was 3) Syllable repetition rate (SRR), which was calculated as (NS-1)/DE (here DE was expressed in seconds to have SRR in syllables per second, number of syllables per echeme (NS) contained all the recognizable syllables of an echeme). Differences between the male calling songs of *S. c. campbelli* and *S. c. gracilis* were analysed by using standardised PCA on the above three acoustic parameters.

### 2.5. Morphometric analysis

**Figure 2:**
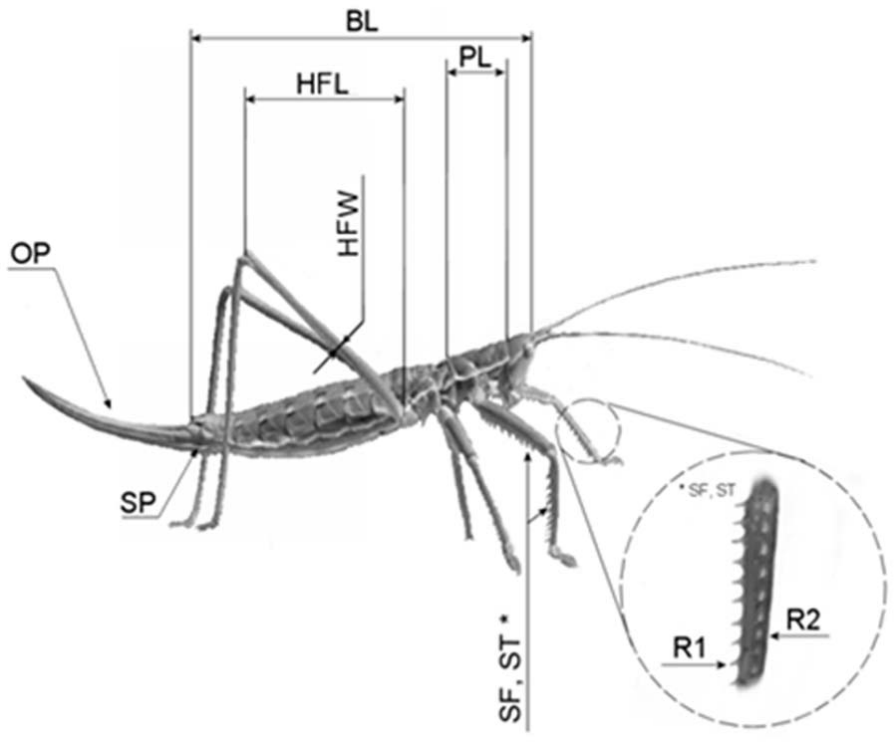
Morphometric parameters measured from the *Saga* species studied. Abbreviations: BL- length of body; HFL - hind femur length; HFW – maximal width of hind femur; OP – ovipositor; PL – dorsal length of the pronotum; SF - average number of spines of the two row of spines on the femur; SP - subgenital plate; and ST – average number of spines of the two row of spines (R1, R2) on the leg.

Morphometric measurements were performed on the three problematic taxa: *S. campbelli campbelli, S. c. gracilis* and *S. pedo*. For the analysis, apart from the ones listed in Table 1, the following additional samples were used: two specimens of *S. c. campbelli* (n = 10), three specimens of *S. c. gracilis* (n = 7) and four dry prepared imagoes of the parthenogenetic bush cricket (two collected; two own rearings; in total n = 9). Parameters used for determining morphological differences between the three taxa were chosen according to those used by Kaltenbach [1] and Kis [39] for distinguishing the species. Principal component analysis (PCA; Statistica 6.0, StatSoft Inc) was run on two log transformed meristic (SF - spines on femur; ST - spines on tibia) and three size-adjusted and log-transformed morphometric characters (HFL - hind femur length; HFW - hind femur width; PL - pronotum length; for the location of these parameters on the specimens see Fig. 2).

Morphometric data were size-adjusted using the method of Senar and colleagues [40] as well as Elliot and colleagues [41]: 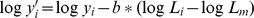, where *y'_i_* is the size-adjusted value of variable y for bush-cricket i, *L_i_* is the body length of bush-cricket i, *L_M_* is the mean body length for all individuals of the three taxa, and *b* is the regression coefficient of log *y* on log *L* using all specimens of a given genotype. For multivariate analyses log *y'* values were used. Since morphometric characters were size-adjusted, body length was left out from further analyses.

Following the PCA, a linear discriminant function analysis (DFA; Statistica 6.0, StatSoft Inc) using a forward stepwise method and based on the Mahalanobis distance was conducted on log-transformed meristic and on size-adjusted and log transformed morphometric data to establish the relative significance of morphological characters in distinguishing among the taxa, but not between sexes. The resultant discriminant functions were used to assign individuals into taxonomic groups. The classification success rate was evaluated on the basis of percent of correctly classified individuals. The relative importance of each character in discriminating genotypes was assessed using the F-to-remove statistic. The graphical representation for the distinction among the taxa was performed by a canonical analysis, and range ellipses around the group centroids were used to visualise relationships between taxa.

Furthermore, two qualitative macroscopic characteristics to distinguish the female specimens of *S. c. campbelli*, *S. c. gracilis* and *S. pedo* were observed: the shape of the subgenital plates, and the ovipositor.

## Results

### 3.1. Phylogenetic analysis of the Saga species showed a monophyletic lineage

**Figure 3:**
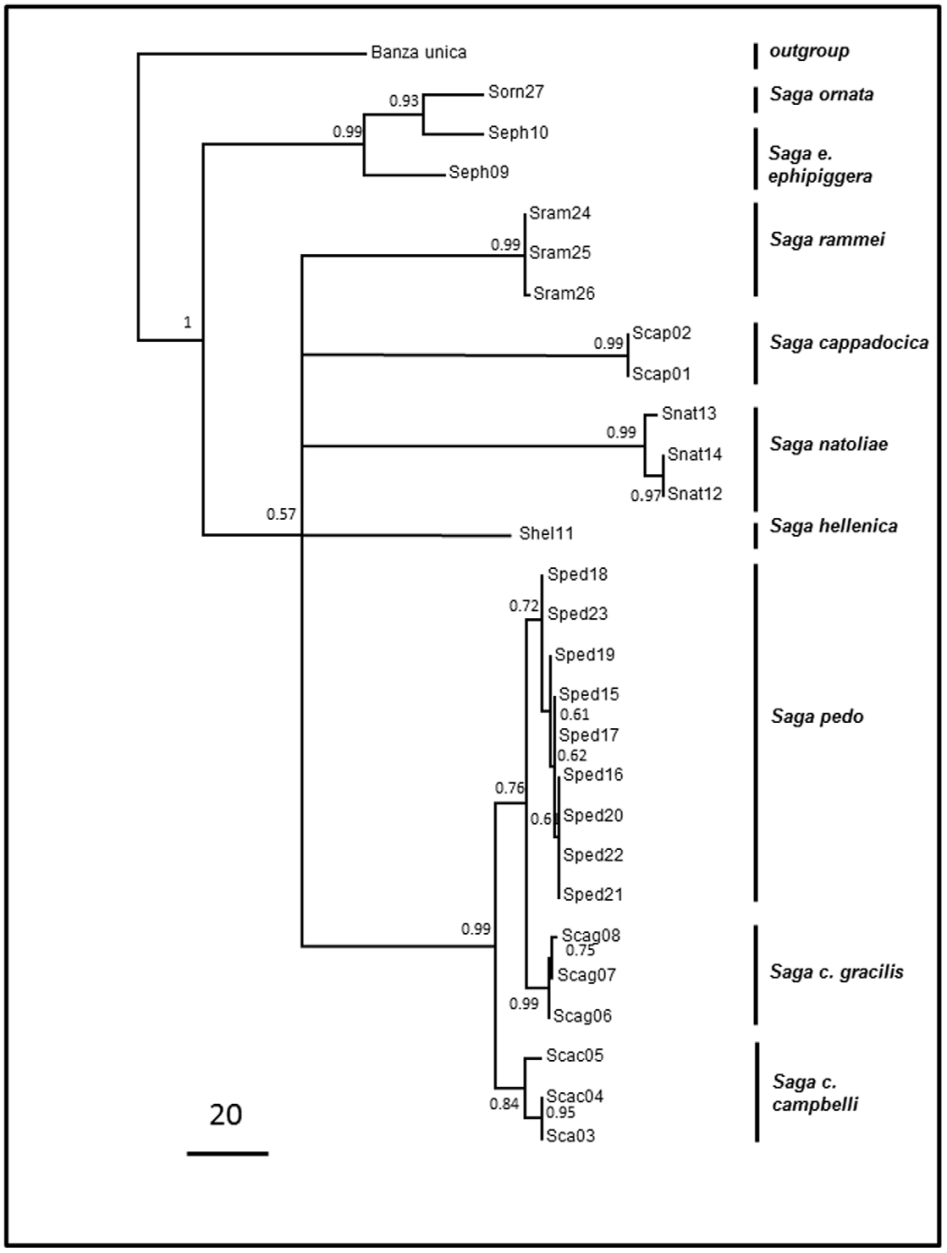
Maximum parsimony tree was inferred from combined mitochondrial (*cytb*, *cox*I, 16SrRNA) DNA sequences of *Saga* species. The mitochondrial consensus tree was inferred from 20 most parsimonious trees (length = 491). Branches corresponding to partitions reproduced in less than 50% trees were collapsed. The consistency index was 0.576, whereas the retention index was0.811. The composite index was 0.496for all sites and 0.468 for the parsimony-informative sites. There were a total of 641 positions in the final dataset, out of which 183 were parsimony informative. Numbers at each node indicate the MP bootstrap values. Vertical bars at right indicate the species. *Banza unica* was designated as outgroup. The MP tree showed that the phylogenetic relationship of *S. pedo* to *S. c. gracilis* is closer than that to *S. c. campbelli.*

Phylogenetic analyses of *cytb*, *cox*I and 16S rRNA genes in eight *Saga* species, using MP or Bayesian inferior analysis based on separated or concatenated data of three loci, showed similar clades, but somewhat different tree topology (see Fig. 3 for the MP tree and Fig. S3 for the Bayesian tree).

The mitochondrial MP tree (Fig. 3) could not fully resolve the relationship of *Saga* species (*cappadocica, natoliae, rammei, hellenica*),nonetheless, it clearly indicates that the *pedo-campbelli* samples are closely related.

The Bayesian analyses of concatenated mitochondrial sequences yielded somewhat different topology compared to the MP tree. Although both methods have placed the basic clade *‘ephippigera-ornata’* closest to the outgroup, Bayesian analyses indicated a clear relationship among the rest of species with high bootstrap support values. Both analyses positioned *B. unica* as an outgroup, underlining the monophyly of the whole sampled Saga genus. Phylogenetic separation of the *‘ephippigera-ornata’* clade and the rest of the European *Saga* species – together with the transitional species, *S. cappadocica*– with the bootstrap value of 1 underline phylogenetic separation of Asian and European *Saga* lineages. Based on the mitochondrial sequences, *S. c. gracilis* was closer to *S. pedo* in the MP tree, while *S. c. gracilis* and *S. c. campbelli* composed a joint line in the Bayesian phylogenetic tree.

**Figure 4:**
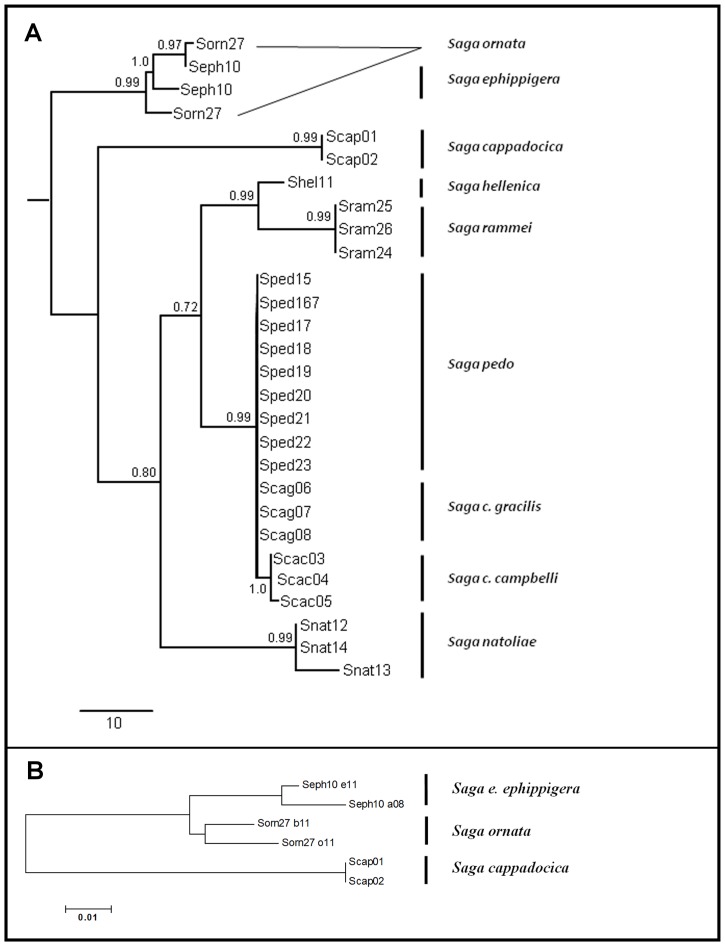
Evolutionary relationship of *Saga* species estimated from the nuclear DNA sequences. Panel A: Maximum Parsimony phylogenetic tree of *Saginae* based on ITS2 sequences. The consistency index was 0.91, whereas the retention index was 0.966 for MP tree. The composite index was 0.888 for all sites and 0.879 for the parsimony-informative sites. There were a total of 829 positions in the final dataset, out of which 95 were parsimony-informative. Vertical bars at right indicate the species. Bootstrap values are presented at each node. The unrooted tree showed the *S. ephippigera-ornata* clade as most basal group. The transient *S. cappadocica* is positioned between the Asian *S. ephippigera-ornata* and the European *Saga* linegae (*S. hellenica, S. rammei, S. pedo, S. c. gracilis, S. c. campbelli, S. natoliae*). Panel B:Asian *Saga* species have a long insertion in their ITS2 sequence, compared to that of European species. Evolutionary relationship of the ITS2 insertions is presented in a Neighbor-Joining tree. The tree is drawn to scale, with branch lengths in the same units as those of the evolutionary distances used to infer the phylogenetic tree. The evolutionary distances were computed using the Maximum Composite Likelihood method and are in the units of the number of base substitutions per site.

The MP and Bayesian analyses based on ITS2 sequences showed a somewhat different tree topology (Fig. 4A). At the species level, the ITS2 locus of *Saga* species appeared to be more conserved than the mitochondrial loci. Both the MP and Bayesian trees shared the same clades, but showed a slightly different tree topology. Both analyses positioned the Asian clades *‘ephippigera-ornata’* and *‘cappadocica’* at the basis of the tree, and confirmed the monophyly of European *Saga* lineage (*natoliae, hellenica, rammei,* and *pedo-campbelli*). The three Asian *Saga* species, *S. cappadocica*, *S.ephippigera* and *S. ornata* samples had a long (155 bp) insertion in their ITS2 sequence, compared to that of European species. Although this insert was removed before the above two trees were built, we have looked at their evolutionary relationship by generating a Neighbour-Joining tree (Fig. S2B). In agreement with the two above trees, the NJ tree has shown that *S. ephippigera* and *S. ornata* were more closely related to each other than to *S. cappadocica*.

Overall comparison of the four phylogenetic trees showed somewhat different topology (Figs. 3 and 4A, and Figs. S3 and S4).On three of the four trees (Figs. 3, 4A, and Fig. S3) the *S. ephippigera – S. ornata* clade appeared to be the most basal group. The *S. rammei* samples clustered together with *S. natoliae* on three trees (Figs. 3, 4A, and Fig. S3), but not on the MP tree generated based on the ITS2 sequences (Fig. 4A) where they shared a branch with *S. hellenica*. The two Asian Minor species, *S. ephippigera* and *S. ornata,* both had two types of ITS2 sequences, showing ancestral ITS2 variability, whereas the European *Saga* species carried only one type of ITS2 sequence. *S. cappadocica, S. hellenica, S. rammei* and *S. natoliae* were positioned as evolutionarily distinct clades, genetically distinctly separated species.

The samples of *S. pedo* clustered onto the same branch with *S. c. campbelli* and *S. c. gracilis* on all four trees. They clustered more closely with *S. c. gracilis* than with *S. c. campbelli* on three of the four trees (Figs. 3, 4A, and Fig. S3), thereby indicating that the phylogenetic relationship of *S. pedo* to *S. c. gracilis* was closer than that to *S. c. campbelli.*

The clade formed by *S. ephippigera* and *S. ornata* appeared to be the most basal one among the *Saga* clades analysed in this study.

### 3.2. Comparative analysis of calling songs indicated substantial differences between the two subspecies of S. campbelli

**Figure 5:**
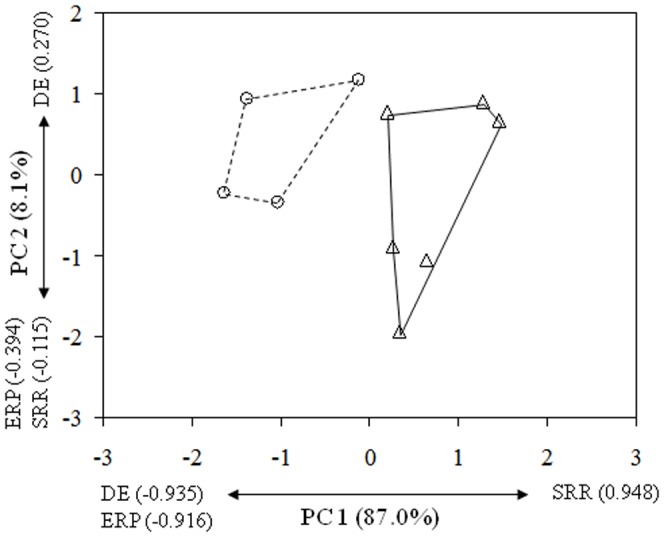
Male calling songs of *Saga campbelli campbelli* (Δ) and *S. c. gracilis* (○) are clearly different. Plots of scores along the first and second principal components (PC) are shown. Variance proportions represented by each PC are indicated together with the factor loadings (correlation). Additional abbreviations: DE – duration of echemes; ERP – echeme repetition period; SRR – syllable repetition rate.

The oscillographic pattern of two subspecies showed a similar basic structure, but conspicuously differed in the measured quantitative rhythmic characters (see Table S2). When male calling songs were compared, the two subspecies *(S. c. campbelli* and *S. c. gracilis*) could be clearly separated based on the PCA. The separation of the two taxa was complete even along the first principal component (PC 1, representing 87% of the total variance) which was correlated highly positively with syllable repetition rate (SRR) and highly negatively with duration of echemes (DE) and echeme repetition period (ERP) (Fig. 5; for dataset see Table S2; for samples of the calling songs see Fig. S5).

### 3.3. Morphometric analysis of S. c. campbelli, S. c. gracilis and S. pedo showed clear differences

**Figure 6:**
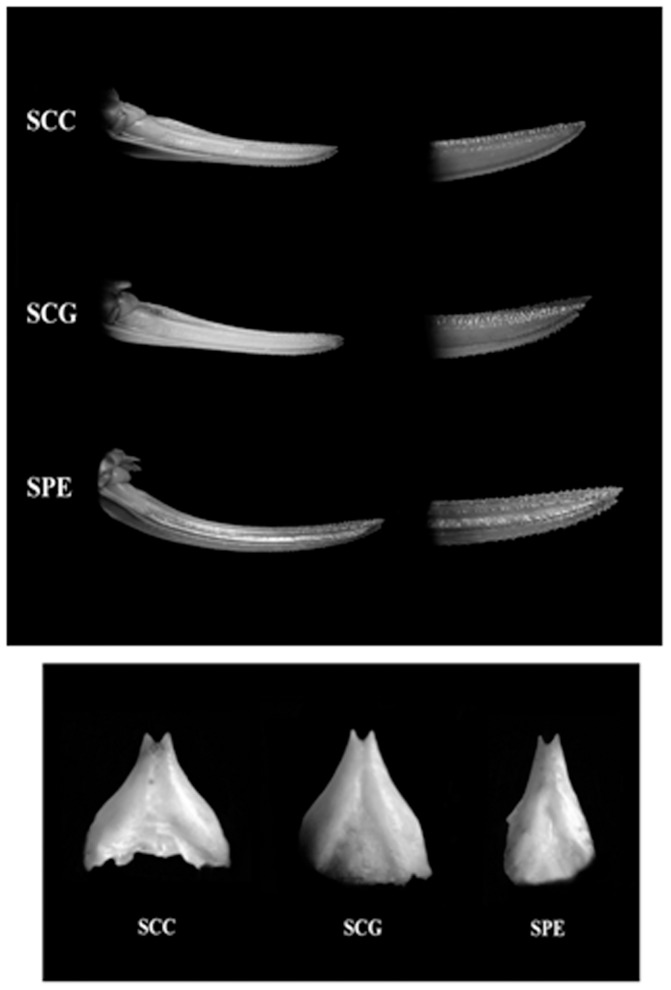
The shape of the ovipositor and subgenital plate is clearly different between *Saga pedo* and *S. c. campbelli*, but not between the two subspecies of the latter. Abbreviations: SCC - *S. c. campbelli;* SCG - *Saga c. gracilis* and SPE – *S. pedo*.

We have analysed female individuals of *Saga campbelli campbelli, S. c. gracilis* and *S. pedo* at the macroscopic level by comparing their morphometric parameters (Fig. 2). Simple visual analysis has shown obvious differences in the shape of the ovipositor and the subgenital plates, between either of the two subspecies and *S. pedo*, respectively. On the other hand, no difference could be seen between *S. c. campbelli* and *S. c. gracilis* at these levels (Fig. 6).

**Figure 7:**
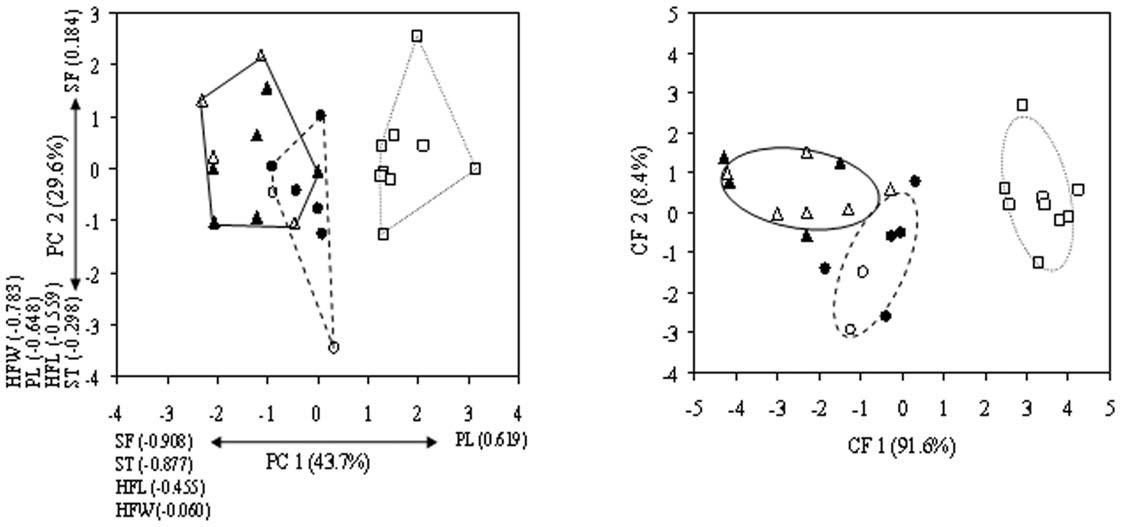
Comparative morphological analysis of *Saga campbelli campbelli, S. c. gracilis,* and *S. pedo* shows clear differences. Labels: Δ - *S. c. campbelli,* ○ - *S. c. gracilis,* and □ - *S. pedo*. Open symbols represent females, whereas filled symbols indicate males. Panel A: Plots of scores along the first and second principal components for morphological characters. Variance proportions represented by each principal component are indicated together with the factor loadings (correlation). Panel B: Canonical analysis based on the three most important morphological characters (ST, HFW and SF) selected by the forward stepwise discriminant function analysis. Sexes were not distinguished in the analysis. Range ellipses are drawn around group centroids, and variance proportions represented by each canonical function are indicated. Abbreviations: CF – canonical function; PC – principal component. For additional abbreviations see the legend of Figure 5.

Based on the PCA, *S. pedo* separated clearly both from *S.c. campbelli* and *S. c. gracilis*, while the latter two showed partial overlap along both first and second principal components (PC; Fig. 7A). PC1 accounted for 43.7% of the total variance and was correlated most positively with PL and most negatively with SF and ST. PC2 accounted for 29.6% of the total variance and was correlated most negatively with HFW and PL.

Forward stepwise discriminant analysis showed that ST (Wilks' λ = 0.20; F-to-remove = 18.85; P<0.001) and HWF (Wilks' λ = 0.12; F-to-remove = 6.74; P = 0.005) both presented significant discriminatory power. However, since SF (Wilks' λ = 0.09; F-to-remove = 3.16; P = 0.063) was also close to statistical significance it was also included to the discriminant function. Based on these characters, all individuals, except one *S. c. campbelli* male (Scag07), were classified correctly. Figure 7B shows three distinguishable clusters, each corresponding to a distinct taxon without any overlap among their range ellipses (for the whole dataset used for morphometric analysis see Table S3).

### 3.4. Genetic distances matrix supported clear interspecific distances among Saga species except between S. pedo and S. c. gracilis

We have also analyzed the uncorrected genetic distances between our *Saga* spp. sequences (Table 2 and Table S4). The mitochondrial distance matrix contained (without the positions, containing gaps and missing data) a total of 1,600 positions, while the ITS2 based contained 829 positions in the final dataset. The largest distance was observed between *S. cappadocica* and *S. ephippigera* (18.66%) with the mitochondrial markers, while with the nuclear marker it amounted to 7.17% between *S. cappadocica* and *S. natoliae*. The smallest distance was observed in the *‘pedo-campbelli’* clade with identical ITS2 sequences of *S. pedo* and *S. c. gracilis*. Interestingly, *S. c. gracilis* was more distantly related to the nominate subspecies, *S. c. campbelli* and the difference amounted to2.62% and 0.28% based on mitochondrial and nuclear markers, respectively. Except among those in the ‘*ephippigera-ornata*’ and in the ‘*pedo-campbelli’* clade(see Table S4), a genetic distance of at least 1% and 10% was observed based on nuclear and mitochondrial markers, respectively, among most of the species. Interestingly, concerning the mitochondrial genes, a larger intra-specific distance - with the maximum of 0.35% - was observed in the parthenogenetic bush cricket (*S. pedo*), than in three of its congeners (see Table S5). The rest of the species (*S. natoliae, S. hellenica, S. rammei,* and *S. cappadocica*) were positioned between the ancient and the evolutionary youngest species (see Table S4). However, the geographical transitional species *S. cappadocica* showed a closer relationship to the Asian species, sharing the 115bp insertion in their ITS2 sequence (Fig. S2 and Fig. 4B).

**Table 2:** Comparison of the genetic and geographic distances among *Saga pedo*, *S. c. campbelli* and *S. c. gracilis*.

	Genetic distance 1^a^	Genetic distance 2^b^	Geographic distance (km)
***S. c. campbelli*** ** - ** ***S. c. gracilis***	**0.0262±0.0016**	**0.0028±0.0006**	**245**
***S. pedo*** ** - ** ***S. c. campbelli***	**0.0275±0.002**	**0.0028±0.0006**	**201–740**
*S. p.* (Hun Bud) - *S. c. c.*	0.0262–0.0309	0.0024–0.0036	732
*S. p.* (Hun Kes) - *S. c. c.*	0.0244–0.0309	0.0024–0.0036	740
*S. p.* (Mac) - *S. c. c.*	0.0262–0.0300	0.0024–0.0036	201
*S. p.* (Bul) - *S. c. c.*	0.0262–0.0300	0.0024–0.0036	478
***S. pedo*** ** - ** ***S. campbelli gracilis***	**0.0142±0.0014**	**0.000**	**295–924**
*S. p.* (Hun Bud) - *S. c. g.*	0.0134–0.0161	0.000	886
*S. p.* (Hun Kes) - *S. c. g.*	0.0107–0.0152	0.000	924
*S. p.* (Mac) - *S. c. g.*	0.0125–0.0143	0.000	434
*S. p.* (Bul) - *S. c. g.*	0.0143–0.0161	0.000	295

a Based on mitochondrial gene sequences (*cox*I, *cytb*, 16S rRNA).

b Based on nuclear gene sequences (ITS2).

## Discussion

The primary aim of this study was to re-visit the evolutionary relationships among European and Asian Minor species of the genus *Saga* and throw light on the origin of the parthenogenetic, tetraploid species, *S. pedo*. We used a comprehensive approach, based on data from molecular, morphological, and song-analytical tools.

### 4.1. Phylogenetic analysis reveals species groups

On the basis of sequence analyses, we have arrived at several findings and conclusions. The most basal group, according to our samplings, is the ‘*ephippigera-ornata*’ clade. The genomes of two Asian Minor species (*S. ephippigera* and *S. ornata*) contain two different ITS2 sequences intra-individually, while all European *Saga* species show the presence of a single ITS2 sequence. The ITS2 region of both Asian Minor species includes a 115 bp insertion (Figure S2 and Fig. 4B), demonstrating their genetic difference from the European species. The geographical transitional species *S. cappadocica,* with a distribution covering the middle of Turkey, also contains the 115 bp insert in its ITS2 locus, on the other hand it has only a single ITS2 sequence. The intra-individual ITS2 variability and the long insertion both appear to be ancient characters of the Asian Minor *Saga* group, while the single ITS2 sequence without the insert is a synapomorphic character of the European *Saga* lineage. The above-mentioned results led us to the conclusion that the European *Saga* lineage originated phylogeographically from the Asian *Saga* group. *S. cappadocica* is a transitional species between Asian and European *Saga* lineages based on the above mentioned molecular findings as well as its geographical distribution.

Mitochondrial MP and Bayesian trees group the *‘ephippigera-ornata’* samples as a basal clade, similarly to the ITS2 trees. However, *S. cappadocica* was placed among the European *Saga* species based on the mitochondrial sequences contradicting the nuclear trees. That may be explained by homoplasy or the geographically transitional living area (the centre of Anatolia) which might have allowed for ancient hybridisation with European *Saga* species.

In earlier analyses performed either by karyological or morphological approaches by others [1], [21], [28], [29], [42] the members of the basal group were not interpreted in a composition that would fit to our molecular data. Based on morphological data, *S. ornata* was placed separately from all its congeners [1], whereas it was considered a basal species - together with *S. cappadocica-* in two karyological studies [21], [23] (Table 3). According to our findings, the transitional *S. cappadocica* was closer to the European *Saga* lineage, the position raised by Kaltenbach [1] on the basis of morphological data(Table 3). The molecular analyses of both the mtDNA and nDNA markers examined confirmed the validity of most morphologically distinguished *Saga* taxa (Table 3). Interestingly, our limited mtDNA sequence data indicate that the *S. ephippigera* samples might include two sibling taxa (Seph09 and Seph10), which are presently treated as a single species (*S. ephippigera*) based on their morphology.

**Table 3:** Comparative data on the phylogenetic relationship of *Saga* species.

Groups	Based on classification	Source
**A: ** ***S. natoliae***_(n = 204)_, ***S. ephippigera***_(n = 209+77)_^1^	morphology	Kaltenbach, 1967
**B:** *S. ornata*_(n = 13)_		
**C:** *S. cappadocica*_(n = 24)_, *S. rammei*_(n = 93)_, *S. c. campbelli*_(n = 27)_, *S. c. gracilis*_(n = 23)_, *S. hellenica*_(n = 58)_		
**D:** *Saga pedo*_(n = 150)_		
***S. ornata***, ***S. cappadocica,*** * S. natoliae*_(n = 1*, n = 1**)_, *S. hellenica*_(n = 1*)_, *S. rhodiensis*_(n = 1**)_, *S. campbelli*_(n = 1*)_, *S. rammei*_(n = 1*)_, *S. pedo*_(n = 1*)_	karyology	Lemmonier-Darcemont et al. (2008**)
*S. pedo*_(n = 6)_ probably originated from *S. rammei* or *S. campbelli*	karyology	Dutrillaux et al. (2009)
**A: ** ***S. ephippigera*** **, ** ***S. ornata*** **, ** ***S. cappadocica***	karyology and acoustic information	Warchalowska et al. (2007*); Warchalowska et al. (2009**)
**B:** *S. natoliae*_(n = 1*)_, *S. rhodiensis*_(n = 1*)_, *S. hellenica*_(n = 3*)_		
**C:** *S. campbelli*_(*S. c. campbelli* n = 5**, *S. c. gracilis* n = 5**)_		
**D:** *S. rammei*_(n = 1**)_		
**E:** *S. pedo*		
**A: ** ***S. ornata***_(*ng* = 1)_, ***S. ephippigera***_(*ng* = 2)_	molecular, acoustic and morphological information	present study
**B:** *S. cappadocia*_(*ng* = 2)_		
**C:** *S. natoliae*_(*ng* = 3)_, *S. hellenica*_(*ng* = 1)_,*S. rammei*_(*ng* = 3)_		
**D:** *S. campbelli*_(*ng* = 3, *nm* = 10, *na* = 4)_, *S. gracilis*_(*ng* = 3; *nm* = 7, *na* = 6)_,*S. pedo*_(*ng* = 9, *nm* = 9)_		

Bold: Most basal species.

Underlined: possible origin species of *S. pedo* ^1^ In this study, data from 209 *Saga e. ephippigera* and 77 *S. e. syriaca* individuals were combined.

*ng*: number of individuals analyzed by genetic tools.

*nm*: number of individuals analyzed by morphological tools.

*na*: number of individuals analyzed by acoustic tools.

### 4.2. The taxonomic position of Saga c. campbelli and Saga c. gracilis should be changed to two separate species based on their morphology, song structure and genetics

We found the male calling songs of *S. c. campbelli* and *S. c. gracilis* to be clearly different regarding the examined quantitative rhythmic song characters (especially the echeme duration and syllable repetition rate; Fig. 5). At the present state of our knowledge, it is difficult to predict whether or not those differences could play a significant role in the premating isolation of the two subspecies if they became sympatric in the future. However, we have to note that the degree and clearness of the difference is surprising and comparable to the difference that can be seen between the songs of well-established species. In contrary to some other bushcricket groups, the songs of European *Saga* species do not seem to bear the most quickly and most conspicuously diverging features between related species (see Heller [43] for a review on the acoustic and morphological divergence patterns in European bush crickets).

Our phylogenetic data show that the ‘*pedo-campbelli*’ monophyletic clade formed by three closely related taxa is well separated from the other *Saga* species. At the same time, the mtDNA analyses support the existence of three separate species within this clade: *S. pedo*, *S. c. gracilis*, and *S. c. campbelli*. The nDNA analyses with more limited variability are congruent with the mtDNA results in this respect. The fact that samples of *S. pedo* and *S. c. gracilis* share the same haplotype indicates a close evolutionary relationship between these two taxa. The *S. c. campbelli* haplotypes differ from the ‘*pedo-gracilis*’ haplotypes by 3 single nucleotide changes only.

Themolecular, song analytical and morphological results described in this study reveal that the current taxonomic status of *S. c. gracilis* – currently a subspecies living in isolated habitats not overlapping with the other subspecies– is not congruent with its phylogenetic position. Our data show distinct (and often significant) differences between *S. c. gracilis* and *S. c. campbelli* on all the above three levels. Therefore, we suggest re-establishing the status of *Saga campbelli* Uvarov, 1921 as a species distinct from *Saga gracilis* Kis, 1962 stat. rev.

Based on the above-mentioned results, the following ten taxa of the genus *Saga* were clustered into the following species groups: A: *S. ephippigera*, *S. ornata;* B: *S. cappadocica;* C: *S. natoliae*, *S. hellenica*, and *S. rammei;* and D: *S. campbelli (S. c. campbelli), S. gracilis (S. c. gracilis),* and *S. pedo* (Table 3).

### 4.3. The possible origin and distribution of Saga pedo

According to their karyotypes, the nine diploid *Saga* species analyzed *[S. ornata, S. ephippigera, S. cappadocica, S. natoliae, S. hellenica, S. campbelli (S. c. campbelli), S. gracilis (S. c. gracilis),* and *S. rammei]* form a group, separated from the parthenogenetic and tetraploid *S. pedo*. While the morphology of *S. pedo* shows similarities with that of *S. campbelli*, according to Warchałowska-Śliwa and colleagues [21], [28], the chromosome set of the former could be derived from that of *S. ephippigera*. According to Lemonnier-Darcemont and colleagues [23], *S. rammei* was found to be the closest to *S. pedo* (see Table 3). They and all other studies on specimen from various localities [22], [24], [25], [26], [27], [44], using different types of cells, have found a karyotype of 4n = 68. Dutrillaux and colleagues [29] investigated the karyotype of *S. pedo* using stromal and neural cells and concluded that it is a pentaploid (5n = 70). They stated that “…chromosomes of roughly similar length and morphology were arbitrarily paired, without presumption of their homology. In some “pairs”, we called a and b the chromosomes which were obviously different.” In another publication written by a largely overlapping set of authors, the karyotype of a bush cricket, *Bradyporus dasypus*, was analyzed by counting both A and B chromosomes without distinguishing them [45]. It is therefore also possible that the samples used by Dutrillaux and colleagues [29] contained B-chromosomes. As commonly accepted, the B chromosomes are supernumerary (accessory) elements of the genome that are usually randomly inherited [46], [47]. Considering the above-mentioned facts, we accepted the tetraploidy of *Saga pedo* until new data will appear showing variation in the karyotype of this species.

In contrast to previous reports, the molecular data described in our manuscript clearly revealed that the parthenogenetic, tetraploid *S. pedo*is derived from a population of *S. gracilis*. This finding is supported by the distribution pattern of the latter species, as it occupies the northernmost territory among the bisexual congeners and occurs sympatric (or even syntopic) with *S. pedo* in the region of Dobrogea (South East Romania). The speciation may have occurred as a result of genome duplication. Polyploidization and the lack of males was caused by parasitoid bacteria (e.g. *Wolbachia pipientis*) in hymenopteran insects [48], furthermore such parasites were recently found in Orthoptera species as well [49]. A similar cause of tetraploidization in the case of *Saga pedo* cannot be excluded. However, Cabrero and colleagues [44] did not find evidence for the presence of *Wolbachia* in this species, similarly to another parthenogenic orthopteran, *Poecilimon intermedius* [50].

Generally, *S. pedo* occupies a range of territories vacant from other congeners, and located towards the North from the ranges of the latter, the compact range reaching southwards on the Balkans about LAT 43°, with a tendency to occur in mountains (above 500 m). Still, a few relict populations of *S. pedo* exist in the mountains of the middle-latitude Balkan Peninsula [2], [51] to the South from LAT 42°, overlapping with the ranges of other *Saga* species. These populations are typically found at altitudes of about 1500–1800 m, connected with xerophyte grass associations on carbonate substrate. Therefore, even when placed within the range of *S. natoliae, S. hellenica, S. rammei,S. campbelli* or *S. gracilis*, the *S. pedo* populations should remain isolated, thus sympatric but never syntopic with the other taxa (the records of *S. natoliae* above 1000 m from Bulgaria are based on misidentifications: Chobanov, unpublished data). Possibly, the same case concerns the co-occurrence of *S. pedo* and *S. ephippigera* in the Caucasus region. These data (together with the geographic area of its range) support the ancient origin of *S. pedo* with periods of Southern expansion and Northern retreats during the glacial/interglacial stages. Today, the only syntopic occurrence of *S. pedo* seems to exist with *S. gracilis* (at least in the region of Southeastern Romania), the species that is most closely related to it both genetically and morphologically.

### 4.4. Prospects for future studies

Our study creates a basis for future investigations of this intriguing group. Complementary studies can now be focused on the evolution of the whole genus *Saga*, involving the species that can be found at the Midle East, and the more distantly related representatives of the subfamilies *Saginae* and *Austrosaginae,* living in Southern Africa and Australia, respectively. Other studies can shed light on the evolution of song, wing reduction and their impact on the distribution of the subfamily.

Concerning the widespread, but patchy distribution of *S. pedo*, further investigations should focus on its dispersal mechanisms, including the examination of the role of predatory birds. Furthermore, the wide distribution range of these animals raises interesting issues about the geographical variability of their reproductive ethology and genetics (i.e. polyploidy), and also the cold tolerance potential of particular xerotherm *Saga* species.

## Supporting Information

Figure S1 **Comparison of the distribution territories of the parthenogenetic** ***S. pedo*** **and those of the bisexual relative species concerned.**
(TIF)

Figure S2 **Asian** ***Saga*** **species have a long insertion of 115 bp in their ITS2 sequence, compared to European species.** The alignment of the ITS2 insertions from three *Saga* species (*S. ornata, S. ephippigera,* and *S. cappadocica*) highlights the differences. In addition, *S. ornata* and *S. ephippigera* also have intra-specific insert variability of their ITS2 sequence.
(DOC)

Figure S3 **Bayesian majority consensus phylogenetic tree of** ***Saginae*** **based on three mitochondrial loci (** ***cytb, coxI, 16S*** **rDNA), assuming the GTR+I+G model of sequence evolution.** Vertical bars at right indicate the species. Posterior probability values are presented at each node. *Banza unica* was used as an outgroup. The Bayesian approach resulted (*pedo*(*gracilis*, *campbelli*)) relationship, while MP analyses showed ((*pedo, gracilis*) *campbelli*).
(TIF)

Figure S4 **Bayesian majority consensus phylogenetic tree of** ***Saginae*** **based on ITS2 gene, assuming the GTR+I+G model of sequence evolution.** Vertical bars at right indicate the species. Posterior probability values are presented at each node. No outgroup was used. Samples of *S. pedo* and *S. c. gracilis* share the same ITS2 sequence, while *S. c. campbelli* differ from *pedo-gracilis* by 3 bp single nucleotide changes.
(TIF)

Figure S5 **Oscillographic pattern of the calling song of the two problematic subspecies.** A)A sequence of three echemes of *S. c. campbelli* (recorded at 26.7°C); B)A sequence of two echemes of *S. c. gracilis* (recorded at 26.3°); C) One echeme of *S. c. campbelli* at a higher time resolution (the second one from oscillogram A); one echeme of *S. c. gracilis* at a higher time resolution (the first echeme in oscillogram B in this figure).
(TIF)

Table S1 **Primers used for the phylogenetic analysis.**
(DOCX)

Table S2 **Acoustic data used in PCA analysis.**
(DOCX)

Table S3 **Morphometric data used in PCA analysis.**
(DOCX)

Table S4 **Inter-specific variability among** ***Saga pedo*** **clones can be higher (even on the same habitat) than that of its bisexual congeners independently from geographic distance.**
(DOCX)

Table S5 **Comparison of the genetic and geographic distances among the** ***Saga*** **species analyzed.**
(DOCX)
