# Correction: Re-Visiting Phylogenetic and Taxonomic Relationships in the Genus *Saga* (Insecta: Orthoptera)

**DOI:** 10.1371/annotation/90f45dc4-e2ab-4727-8278-2787c215bc61

**Published:** 2012-11-12

**Authors:** Balázs Kolics, Zoltán Ács, Dragan Petrov Chobanov, Kirill Márk Orci, Lo Shun Qiang, Balázs Kovács, Előd Kondorosy, Kincső Decsi, János Taller, András Specziár, László Orbán, Tamás Müller

The following information was missing from the Funding section: Bioacoustic examination was supported by a research grant of the Hungarian Scientific Research Fund (OTKA K81929). 

