# Supplementary figures and images for: Re-Visiting Phylogenetic and Taxonomic Relationships in the Genus Saga (Insecta: Orthoptera)
Source: PLoS One. 2012 Aug 10;7(8):e42229. doi: 10.1371/journal.pone.0042229 (PMC3420257; doi:10.1371/journal.pone.0042229)

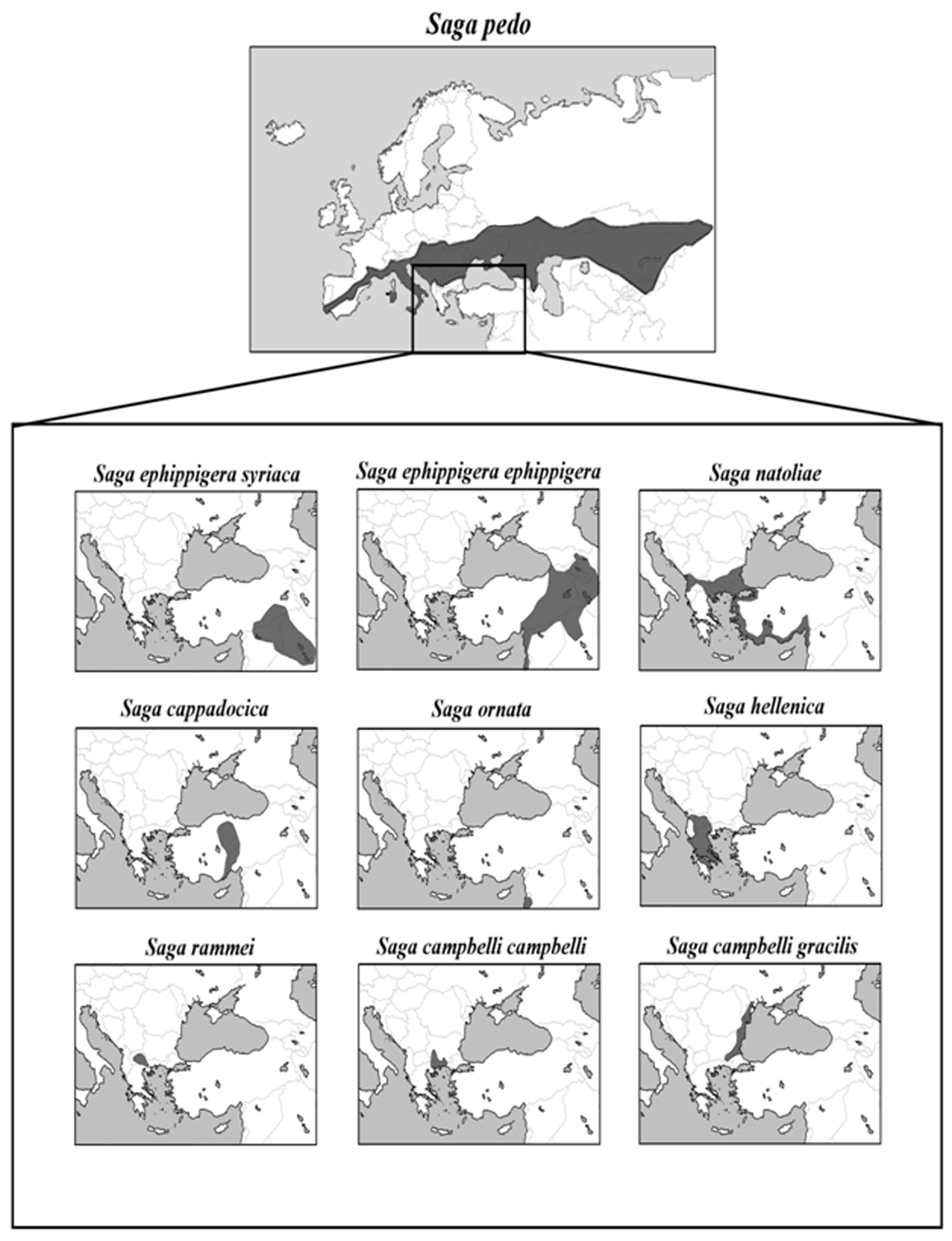

Supplement: Figure S1 — Comparison of the distribution territories of the parthenogenetic S. pedo and those of the bisexual relative species concerned. (TIF) [file pone.0042229.s001.tif]

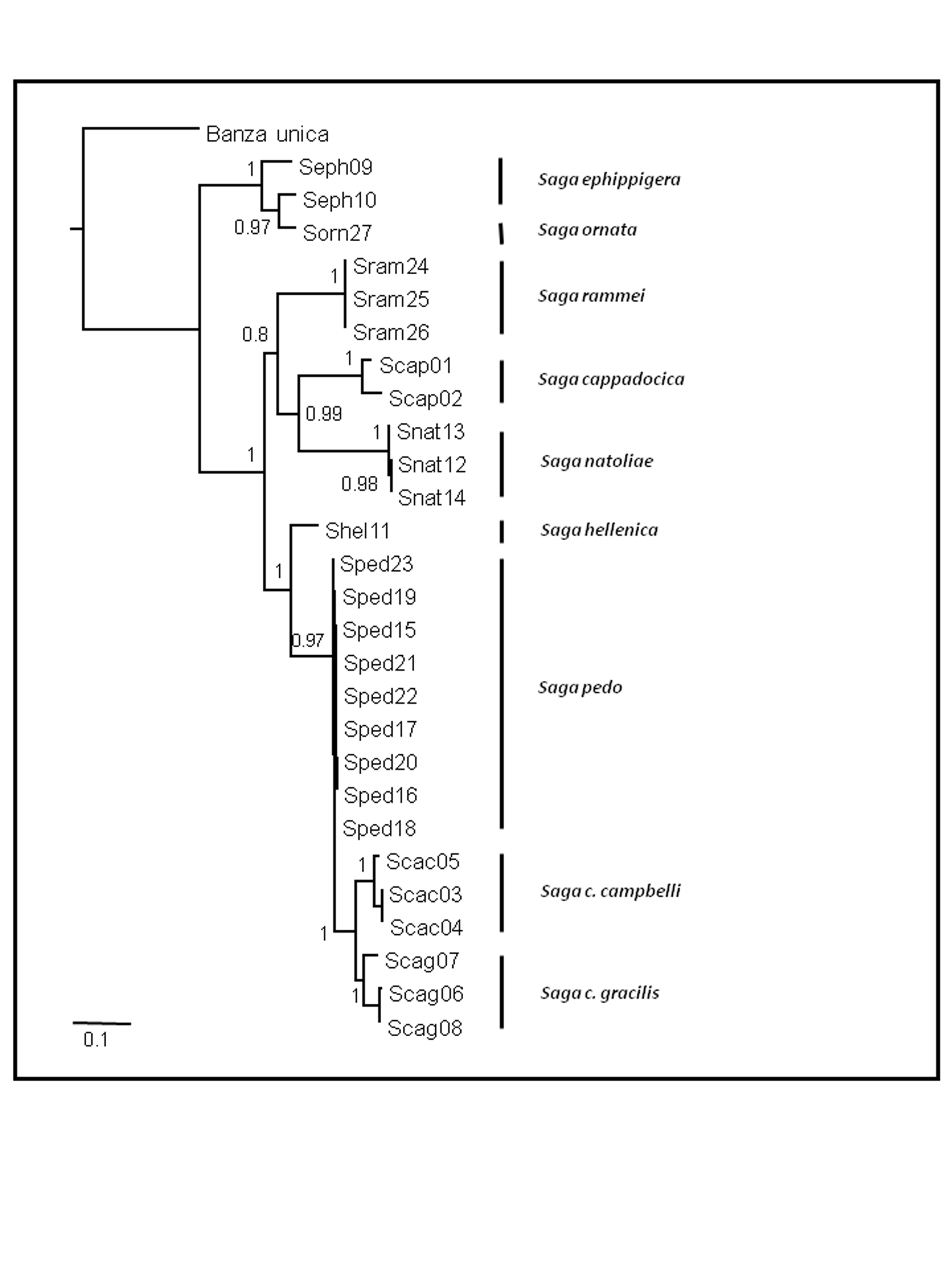

Supplement: Figure S3 — Bayesian majority consensus phylogenetic tree of Saginae based on three mitochondrial loci ( cytb, coxI, 16S rDNA), assuming the GTR+I+G model of sequence evolution. Vertical bars at right indicate the species. Posterior probability values are presented at each node. Banza unica was used as an outgroup. The Bayesian approach resulted (pedo(gracilis, campbelli)) relationship, while MP analyses showed ((pedo, gracilis) campbelli). (TIF) [file pone.0042229.s003.tif]

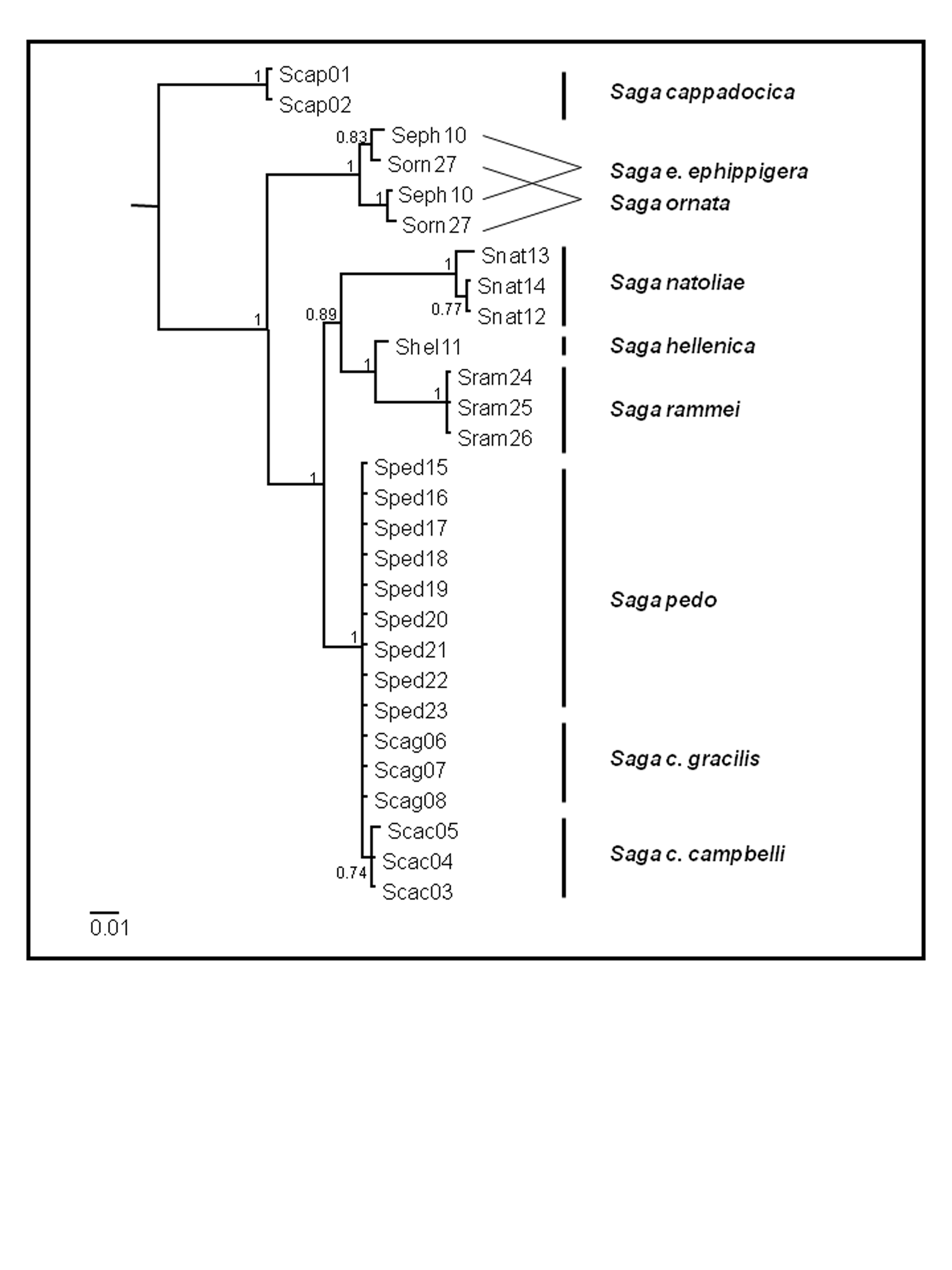

Supplement: Figure S4 — Bayesian majority consensus phylogenetic tree of Saginae based on ITS2 gene, assuming the GTR+I+G model of sequence evolution. Vertical bars at right indicate the species. Posterior probability values are presented at each node. No outgroup was used. Samples of S. pedo and S. c. gracilis share the same ITS2 sequence, while S. c. campbelli differ from pedo-gracilis by 3 bp single nucleotide changes. (TIF) [file pone.0042229.s004.tif]
