# Supplementary material for: Re-Visiting Phylogenetic and Taxonomic Relationships in the Genus Saga (Insecta: Orthoptera)
Source: PLoS One. 2012 Aug 10;7(8):e42229. doi: 10.1371/journal.pone.0042229 (PMC3420257; doi:10.1371/journal.pone.0042229)
Supplement: Figure S2 — Asian Saga species have a long insertion of 115 bp in their ITS2 sequence, compared to European species. The alignment of the ITS2 insertions from three Saga species (S. ornata, S. ephippigera, and S. cappadocica) highlights the differences. In addition, S. ornata and S. ephippigera also have intra-specific insert variability of their ITS2 sequence. (DOC) [file pone.0042229.s002.doc]

Supplementary Figure S2.

Sorn27_b11 CGGGGAGCTTCGTCTCCCCGTTCGACGCCGAGCCTTGTCGCTCAGGCGGTTGGTGAGACC 60

Sorn27_o11 CGGGGAGCTTCGTCTCCCCGTTCGACGCCGAGCCTTGTCGCTCAGGCGGTTGGTGAGACC 60

Seph10_e11 CGGGGAGCTTTGTCTCCCCGTTCGACGCCGAGCCCTGTAGCTCCGGCGGTTGGTGAGACC 60

Seph10_a08 CGGGGAGCTTTGTCTCCCCGTTCGACGCCGAGCCCTGTAGCTCCGGCGGTTGGTGAGACC 60

Scap01 CGGGGAGCATCGTCTACCCATTCGCCGCCGAGCGCTGTCGCTCAGGCGGTTGGTGAGACC 60

Scap02 CGGGGAGCATYGTCTACCCATTCGCCGCCGAGCGCTGTCGCTCAGGCGGTTGGTGAGACC 60

******** * **** *** **** ******** *** **** ****************

Sorn27_b11 TATCGGTCTCGATGGCGTGGGCATCGCTTCGGCGTCCCGCGGGTGCGAGCACAGG 115

Sorn27_o11 TCTCGGTCTCGATGGCGTGGGCATCGCTTCGGCGTCCCGCAAGTGCGAGCACAGG 115

Seph10_e11 TCTCGGTCTCGATGGCGTGGGCATCGCTTCGGCGTCCCGCAGGTGCGAGCACAGG 115

Seph10_a08 TCTAGGTCTCGATGGCGTGGGCATCGCTTCGGCGTCCCGCGGGTGCGAGCACAGG 115

Scap01 TGTCGGTCTCGATGGCGTGGGCATCGCTTCGGCGTCCCGCTGTTGCGCGTCCAGG 115

Scap02 TGTCGGTCTCGATGGCGTGGGCATCGCTTCGGCGTCCCGCTGTTGCGCGTCCAGG 115

* * ************************************ **** * ****
