# Supplementary material for: Re-Visiting Phylogenetic and Taxonomic Relationships in the Genus Saga (Insecta: Orthoptera)
Source: PLoS One. 2012 Aug 10;7(8):e42229. doi: 10.1371/journal.pone.0042229 (PMC3420257; doi:10.1371/journal.pone.0042229)
Supplement: Table S1 — Primers used for the phylogenetic analysis. (DOCX) [file pone.0042229.s006.docx]

| **Gene** | **Primer** | **Sequence 5'-3'** | **Reference** |
| --- | --- | --- | --- |
| *cox*I | LCO1490 | GGTCAACAAATCATAAAGATATTGG | Folmer et al. (1994) |
| *cox*I | HCO2198 | TAAACTTCAGGGTGACCAAAAAATCA | Folmer et al. (1994) |
| *cytb* | CytbP21c | CCATCCAACATCTCAGCATGATGAAA | Huang et al. (2000) |
| *cytb* | Cytb2Rc | CCWARTTTATTAGGAATAGATCG | Huang et al. (2000) |
| 16S rRNA | 16Sa | CGCCTGTTTATCAAAAACAT | Palumbi et al. (1991) |
| 16S rRNA | 16Sb | CTCCGGTTTGAACTCAGATCA | Palumbi et al. (1991) |
| ITS2 | ITSfw | TCCTCCGCTTATTGATATGC | this study |
| ITS2 | ITSrev | GGAAGTAAAAGTCGTAACAAGG | this study |
