# Supplementary material for: Re-Visiting Phylogenetic and Taxonomic Relationships in the Genus Saga (Insecta: Orthoptera)
Source: PLoS One. 2012 Aug 10;7(8):e42229. doi: 10.1371/journal.pone.0042229 (PMC3420257; doi:10.1371/journal.pone.0042229)
Supplement: Table S2 — Acoustic data used in PCA analysis. (DOCX) [file pone.0042229.s007.docx]

| Species | Specimen | DE | ERP | SRR |
| --- | --- | --- | --- | --- |
| *Saga campbelli campbelli* | Scac05 | 2298 | 14997 | 34.3 |
|  | Scac32 | 1904 | 14275 | 34.4 |
|  | Scac33 | 1770 | 15340 | 33.2 |
|  | Scac34 | 1871 | 11588 | 36.1 |
|  | Scac35 | 1638 | 11354 | 34.1 |
|  | Scac36 | 2313 | 13508 | 32.5 |
| *Saga campbelli gracilis* | Scag08 | 3027 | 16840 | 31.4 |
|  | Scag37 | 3377 | 16418 | 30.3 |
|  | Scag38 | 3081 | 17536 | 28.9 |
|  | Scag39 | 2645 | 13888 | 32.2 |
