# Supplementary material for: Re-Visiting Phylogenetic and Taxonomic Relationships in the Genus Saga (Insecta: Orthoptera)
Source: PLoS One. 2012 Aug 10;7(8):e42229. doi: 10.1371/journal.pone.0042229 (PMC3420257; doi:10.1371/journal.pone.0042229)
Supplement: Table S3 — Morphometric data used in PCA analysis. (DOCX) [file pone.0042229.s008.docx]

| Species | Sex | SF | ST | HFL | HFW | PL | L |
| --- | --- | --- | --- | --- | --- | --- | --- |
| *Saga campbelli gracilis* | male | 13 | 13 | 37 | 1.9 | 8.1 | 47 |
|  | male | 10 | 13 | 38 | 1.6 | 7.0 | 40 |
|  | male | 12 | 12 | 39 | 1.8 | 8.9 | 55 |
|  | male | 11 | 13 | 41 | 1.9 | 8.9 | 50 |
|  | male | 11 | 13 | 37 | 2.1 | 8.0 | 46 |
|  | female | 11 | 14 | 40 | 2.4 | 10.0 | 51 |
|  | female | 13 | 12 | 40 | 2.2 | 9.1 | 56 |
| *Saga campbelli campbelli* | female | 14 | 14 | 39 | 1.8 | 8.0 | 52 |
|  | female | 13 | 14 | 41 | 1.7 | 8.7 | 53 |
|  | female | 13 | 12 | 38 | 1.7 | 8.0 | 54 |
|  | female | 11 | 14 | 40 | 2.1 | 9.2 | 55 |
|  | male | 12 | 14 | 41 | 1.8 | 8.8 | 48 |
|  | male | 13 | 13 | 42 | 1.8 | 8.0 | 48 |
|  | male | 12 | 13 | 38 | 1.7 | 7.5 | 46 |
|  | male | 12 | 13 | 41 | 1.7 | 8.5 | 52 |
|  | male | 11 | 12 | 40 | 1.8 | 9.0 | 53 |
|  | male | 13 | 14 | 43 | 1.8 | 9.0 | 52 |
| *Saga pedo* | female | 10 | 11 | 42 | 2.3 | 10.0 | 57 |
|  | female | 10 | 11 | 41 | 2.1 | 9.9 | 58 |
|  | female | 10 | 11 | 42 | 2.0 | 10.0 | 58 |
|  | female | 10 | 11 | 40 | 1.9 | 9.4 | 56 |
|  | female | 10 | 11 | 41 | 2.2 | 10.0 | 61 |
|  | female | 10 | 11 | 38 | 1.7 | 9.5 | 58 |
|  | female | 10 | 10 | 40 | 2.1 | 12.0 | 62 |
|  | female | 10 | 10 | 37 | 1.8 | 8.7 | 50 |
|  | female | 11 | 10 | 42 | 2.1 | 10.0 | 58 |
