# Supplementary material for: Re-Visiting Phylogenetic and Taxonomic Relationships in the Genus Saga (Insecta: Orthoptera)
Source: PLoS One. 2012 Aug 10;7(8):e42229. doi: 10.1371/journal.pone.0042229 (PMC3420257; doi:10.1371/journal.pone.0042229)
Supplement: Table S4 — Inter-specific variability among Saga pedo clones can be higher (even on the same habitat) than that of its bisexual congeners independently from geographic distance. (DOCX) [file pone.0042229.s009.docx]

| **Conspecific individuals** | **Genetic distance 1^a^** | **Genetic distance 2^b^** | **Geographic distance (km)** |
| --- | --- | --- | --- |
| ***S. cappadocica - S. cappadocica*** | 0.0000 | 0.0000 | 0 |
| ***S. campbelli campbelli - S. c. c.*** | 0.0000 - 0.0134 | 0.0000 - 0.0012 | 0 |
| ***S. campbelli gracilis - S. c. g.*** | 0.0009 - 0.0018 | 0.0097 | 245 |
| ***S. rammei - S. rammei* ^2^** | 0.0000 - 0.0009 | 0.0000 | 17 |
| ***S. natoliae - S. natoliae* ^3^** | 0.0000 - 0.0062 | 0.0000 - 0.0073 | 110 |
| ***S. ornata - S. ornata*** | - | 0.0122 | 0 |
| ***S. ephippigera* (Tur) *-* *S. ephippigera* (Syr)** | 0.0699 | - | 622 |
| ***S. pedo - S. pedo* (summarised) ^1^** | 0.0000 - 0.0035 | 0.0000 | 144 - 963 |
| *S. pedo* (Hun Bud) - *S. pedo* (Hun Bud) | 0.0009 | 0.0000 | 0 |
| *S. pedo* (Hun Bud) - *S. pedo* (Hun Kes) | 0.0000 - 0.0035 | 0.0000 | 144 |
| *S. pedo* (Hun Bud) - *S. pedo* (Mac) | 0.0027 - 0.0035 | 0.0000 | 744 |
| *S. pedo* (Hun Bud) - *S. pedo* (Bul) | 0.0000 - 0.0009 | 0.0000 | 882 |
| *S. pedo* (Hun Kes) - *S. pedo* (Hun Kes) | 0.0009 - 0.0027 | 0.0000 | 0 |
| *S. pedo* (Hun Kes) - *S. pedo* (Mac) | 0.0027 | 0.0000 | 713 |
| *S. pedo* (Hun Kes) - *S. pedo* (Bul) | 0.0009 - 0.0035 | 0.0000 | 963 |
| *S. pedo* (Bul) - *S. pedo* (Bul) | 0.0000 | 0.0000 | 0 |
| *S. pedo* (Mac) - *S. pedo* (Bul) | 0.0035 | 0.0000 | 679 |
