# Supplementary material for: Re-Visiting Phylogenetic and Taxonomic Relationships in the Genus Saga (Insecta: Orthoptera)
Source: PLoS One. 2012 Aug 10;7(8):e42229. doi: 10.1371/journal.pone.0042229 (PMC3420257; doi:10.1371/journal.pone.0042229)
Supplement: Table S5 — Comparison of the genetic and geographic distances among the Saga species analyzed. (DOCX) [file pone.0042229.s010.docx]

|  | | **Genetic distance 1^a^** | **Genetic distance 2^b^** | **Geographic distance (km)** |
| --- | --- | --- | --- | --- |
| ***S. pedo*** | *S. c. gracilis* | 0.0142 ± 0.0014 | 0.0000 ± 0.0000 | 295 – 924 |
|  | *S. c. campbelli* | 0.0275 ± 0.0020 | 0.0028 ± 0.0006 | 201 – 478 |
|  | *S. hellenica* | 0.1024 ± 0.0010 | 0.0233 ± 0.0000 | 113 – 653 |
|  | *S. rammei* | 0.1243 ± 0.0017 | 0.0321 ± 0.0000 | 108 – 692 |
|  | *S. cappadocica* | 0.1357 ± 0.0008 | 0.0604 ± 0.0000 | 740 – 1690 |
|  | *S. natoliae* | 0.1494 ± 0.0020 | 0.0418 ± 0.0031 | 124 – 764 |
|  | *S. ornata* | 0.1609 ± 0.0016 | 0.0467 ± 0.0007 | 1327 – 2208 |
|  | *S. ephippigera* (Tur) | 0.1615 ± 0.0018 | 0.0447 ± 0.0000 | 1056 – 2018 |
|  | *S. ephippigera* (Syr) | 0.1628 ± 0.0021 | 0.0473 ± 0.0000 | 1322 – 2201 |
| ***S. campbelli gracilis*** | *S. c. campbelli* | 0.0262 ± 0.0016 | 0.0028 ± 0.0006 | 245 |
|  | *S. hellenica* | 0.1060 ± 0.0006 | 0.0233 ± 0.0000 | 390 |
|  | *S. rammei* | 0.1300 ± 0.0011 | 0.0321 ± 0.0000 | 336 – 353 |
|  | *S. cappadocica* | 0.1391 ± 0.0010 | 0.0604 ± 0.0000 | 807 |
|  | *S. natoliae* | 0.1478 ± 0.0009 | 0.0418 ± 0.0032 | 231 – 336 |
|  | *S. ornata* | 0.1654 ± 0.0007 | 0.0467 ± 0.0007 | 1282 |
|  | *S. ephippigera* (Tur) | 0.1565 ± 0.0007 | 0.0447 ± 0.0000 | 1157 |
|  | *S. ephippigera* (Syr) | 0.1638 ± 0.0007 | 0.0473 ± 0.0000 | 1277 |
| ***S. campbelli campbelli*** | *S. hellenica* | 0.1047 ± 0.0013 | 0.0262 ± 0.0008 | 145 |
|  | *S. rammei* | 0.1329 ± 0.0014 | 0.0350 ± 0.0007 | 92 – 108 |
|  | *S. cappadocica* | 0.1363 ± 0.0048 | 0.0621 ± 0.0007 | 1050 |
|  | *S. natoliae* | 0.1472 ± 0.0024 | 0.0423 ± 0.0033 | 32 – 92 |
|  | *S. ornata* | 0.1610 ± 0.0020 | 0.0484 ± 0.0010 | 1488 |
|  | *S. ephippigera* (Tur) | 0.1555 ± 0.0006 | 0.0464 ± 0.0008 | 1400 |
|  | *S. ephippigera* (Syr) | 0.1603 ± 0.0006 | 0.0489 ± 0.0008 | 1480 |
| ***S. rammei*** | *S. hellenica* | 0.1154 ± 0.0006 | 0.0171 ± 0.0000 | 45 – 57 |
|  | *S. cappadocica* | 0.1299 ± 0.0006 | 0.0750 ± 0.0000 | 1140 – 1155 |
|  | *S. natoliae* | 0.1503 ± 0.0008 | 0.0483 ± 0.0033 | 0 – 125 |
|  | *S. ornata* | 0.1665 ± 0.0007 | 0.0649 ± 0.0008 | 1567 – 1576 |
|  | *S. ephippigera* (Tur) | 0.1546 ± 0.0000 | 0.0629 ± 0.0000 | 1490 – 1506 |
|  | *S. ephippigera* (Syr) | 0.1687 ± 0.0007 | 0.0655 ± 0.0000 | 1557 – 1568 |
| ***S. hellenica*** | *S. cappadocica* | 0.1265 ± 0.0000 | 0.0670 ± 0.0000 | 1195 |
|  | *S. natoliae* | 0.1453 ± 0.0001 | 0.0418 ± 0.0037 | 56 – 166 |
|  | *S. ornata* | 0.1755 | 0.0558 ± 0.0009 | 1620 |
|  | *S. ephippigera* (Tur) | 0.1582 | 0.0538 | 1545 |
|  | *S. ephippigera* (Syr) | 0.1727 | 0.0564 | 1613 |
| ***S. cappadocica*** | *S. natoliae* | 0.1466 ± 0.0011 | 0.0717 ± 0.0035 | 1030 – 1140 |
|  | *S. ornata* | 0.1793 | 0.0689 ± 0.0008 | 658 |
|  | *S. ephippigera* (Tur) | 0.1738 | 0.0683 ± 0.0000 | 654 |
|  | *S. ephippigera* (Syr) | 0.1866 | 0.0682 ± 0.0000 | 353 |
| ***S. natoliae*** | *S. ornata* | 0.1788 ± 0.0013 | 0.0612 ± 0.0037 | 1460 – 1567 |
|  | *S. ephippigera* (Tur) | 0.1624 ± 0.0015 | 0.0586 ± 0.0038 | 1381 – 1490 |
|  | *S. ephippigera* (Syr) | 0.1784 ± 0.0013 | 0.0625 ± 0.0038 | 1452 - 1557 |
| ***S. ornata*** | *S. ephippigera* (Tur) | 0.0708 | 0.0061 ±0.0069 | 621 |
|  | *S. ephippigera* (Syr) | 0.0386 | 0.0097 ±0.0017 | 8 |
| ***S. ephippigera (Tur)*** | *S. ephippigera* (Syr) | 0.0699 | 0.0097 | 621 |
